# Intestinal perforation after nivolumab immunotherapy for a malignant melanoma: a case report

**DOI:** 10.1186/s40792-017-0370-7

**Published:** 2017-08-25

**Authors:** Koji Yasuda, Toshiaki Tanaka, Soichiro Ishihara, Kensuke Otani, Takeshi Nishikawa, Tomomichi Kiyomatsu, Kazushige Kawai, Keisuke Hata, Hiroaki Nozawa, Yuri Masui, Yukako Shintani, Toshiaki Watanabe

**Affiliations:** 1 Department of Surgical Oncology, Faculty of Medicine, University of Tokyo Hospital, 7-3-1 Hongo, Bunkyo-ku, Tokyo, 113-0033 Japan; 2 Department of Dermatology, Faculty of Medicine, The University of Tokyo, Tokyo, Japan; 3 Department of Pathology, Faculty of Medicine, The University of Tokyo, Tokyo, Japan

**Keywords:** Nivolumab, Intestinal perforation, Malignant melanoma

## Abstract

**Background:**

Nivolumab is a monoclonal antibody against programmed death 1 and has become a standard treatment of advanced melanoma because of its durable response and survival benefits. In this report, we present a case of severe intestinal perforation after nivolumab immunotherapy for malignant melanoma.

**Case presentation:**

A 73-year-old man with stage IV malignant melanoma underwent nivolumab therapy. The patient presented to our hospital because of a progressing abdominal pain. Radiological evaluation revealed evidence of free intraperitoneal air. Therefore, we diagnosed the patient as having an intestinal perforation, which was successfully resolved after surgical treatment.

**Conclusion:**

Although intestinal perforation after nivolumab immunotherapy is rare, it can be severe and requires early diagnosis and emergency surgery to ensure a favorable prognosis.

## Background

Immune checkpoint blockade is a new therapy that uses cytotoxic T-lymphocyte antigen 4 (CTLA-4) and programmed death 1 (PD-1) inhibitors to treat melanoma and squamous cell lung cancer. In the last decade, studies have clarified the integral role of the immune regulatory pathway involving PD-1 (a receptor expressed in activated T and B cells) and PD-1 ligands (PD-L1 and PD-L2) in the downregulation of antitumor immunity. Thus, inhibition of this immune regulatory pathway by using blocking monoclonal antibodies (mAbs) against PD-1 or PD-L1 is emerging as an effective therapy for achieving tumor regression in patients with advanced disease [1]. Nivolumab is an anti-PD-1 mAb that provides a durable response in various advanced malignancies [2, 3]. For example, in cases of melanoma, nivolumab provides 1- and 2-year overall survival rates of 62 and 43%, respectively [4]. Thus, nivolumab has recently become a standard treatment for patients with advanced melanoma [5].

However, the use of immune checkpoint inhibitors can lead to novel autoimmune-related adverse events, including interstitial pneumonia, colitis, vitiligo, autoimmune hepatitis, and endocrine dysfunction [4, 6]. In this report, we describe a case of advanced melanoma with intestinal perforation that developed shortly after the start of nivolumab therapy. The patient underwent surgical treatment for the intestinal perforation and medical management of sepsis and recovered successfully without complications. The patient provided informed consent for publishing this report.

## Case presentation

**Fig. 1:**
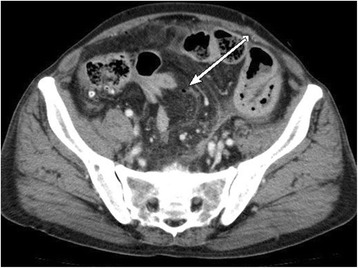
Computed tomographic findings: abdominal and pelvic computed tomography scan showing free air bubbles near the small intestine

A malignant melanoma in the anal canal was treated with curative surgery when the patient was 72 years of age. Five months after surgery, curative lymphadenectomy was performed owing to the recurrence of metastases in the right inguinal and right lateral lymph nodes. Fourteen months after the initial curative surgery, when the patient was 73 years of age, multiple metastases were observed in the lungs, liver, and bones, thyroid gland, and subcutaneous tissue; we accordingly initiated nivolumab treatment. We treated the patient with intravenous nivolumab therapy (2 mg/kg every 3 weeks), but he subsequently developed abdominal distension and progressive diffuse abdominal pain within a week after receiving his third dose of nivolumab. Abdominal and pelvic computed tomography revealed free air near the small intestine (Fig. 1). Thus, the patient was hospitalized under the diagnosis of intestinal perforation. His medical history included diabetes mellitus, pericarditis, and previous surgery (laparoscopic abdominoperineal resection melanoma in the anal canal, and resection of lateral and inguinal lymph node metastases).

Initial evaluation revealed dehydration and tachycardia (97 beats/min), a low-grade fever, abdominal distension, tenderness on palpation, no peristalsis, rebound phenomenon, and muscle stiffness. Laboratory testing revealed the following results: white blood cells, 7100/μL; hemoglobin, 13.7 g/dL; platelets, 190,000/μL; creatinine, 0.55 mg/dL; and C-reactive protein, 0.05 mg/L. Based on these findings, he underwent emergency surgery, during which purulent free fluid, no necrosis of the colon and small intestine, and extensive adhesion of the small intestine with perforation of the small intestine were found. The perforation was found in the ileum, 240 cm away from the Treitz ligament. The patient had generalized peritonitis and underwent an emergency operation in a poor general condition; partial resection of the small intestine including the perforation area was performed. We decided not to perform intestinal anastomosis but to perform ileostomy after considering the risk of sutural insufficiency. Therefore, we performed small intestinal resection with ileostomy and cavity lavage. During the surgery, extensive adhesion caused by past surgery was found in the abdominal cavity of the patient. The intestinal wall distal from the perforation area was injured during surgery; partial resection of the small intestine including the injured area was required. We accordingly resected 50 cm of the small intestine. No surgical complications occurred during the perioperative period, although the patient was admitted to the intensive care unit because of hemodynamic instability. During the postoperative period, the patient exhibited signs of systemic inflammation and was prescribed several antibiotics, which included vancomycin hydrochloride. The patient required mechanical ventilation on the fifth postoperative day, and the patient’s hemodynamic status remained stable without the need for vasopressors. Thus, we maintained the intermediate therapy. The patient had a bowel movement on the tenth postoperative day, and oral administration of water was initiated, which he tolerated well. The patient was discharged at 42 days after the surgery, with no complications, with adequate gastrointestinal function, and with the ability to tolerate an oral diet.

**Fig. 2:**
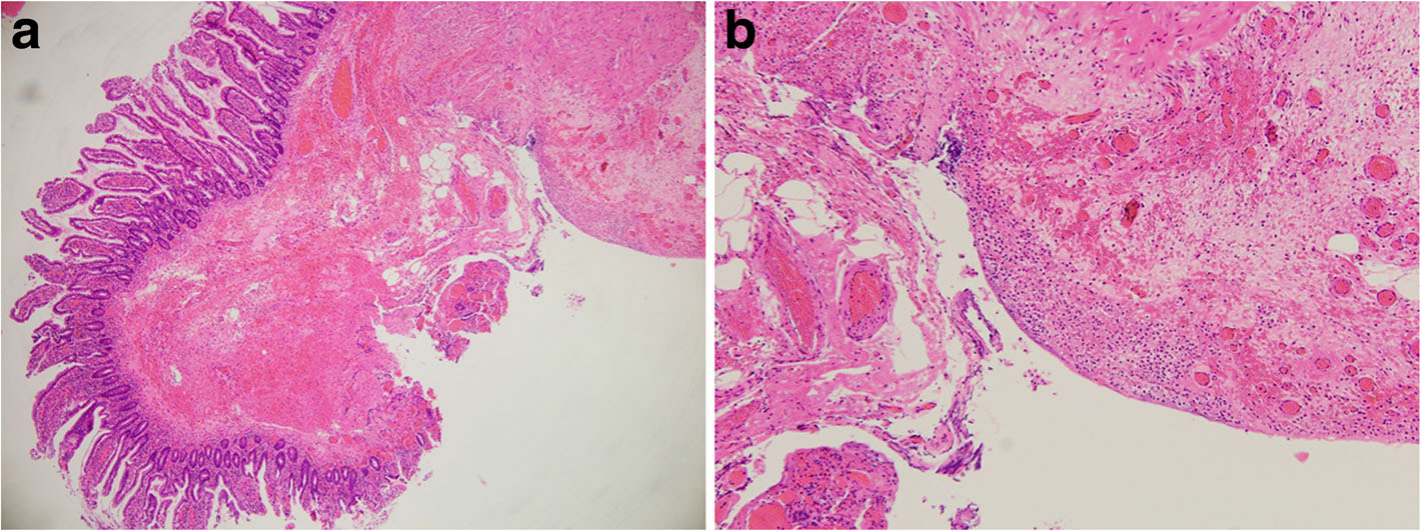
Histopathological findings: histopathological examination results showing neutrophil infiltration and fibrinous deposits near the perforation. **a** High-power field. **b** Low-power field

Macroscopic evaluation of the intestinal mucosal membrane revealed a brown, white, and moss-like appearance on the serosal side. We also identified a perforation site that was 14 cm from the oral margin and 35 cm from the anal margin. Histopathological examination revealed neutrophil infiltration and fibrinous deposits near the perforation site (Fig. 2). Inflammations were more severe in the stromal areas around the perforation than in other areas. The non-perforated section of the intestinal tract had a normal luminal structure, and we did not detect any tumorous lesions on mucous membrane, or blood clots. We did not recognize the intestinal metastasis before nivolumab therapy by CT and PET examination.

## Discussion

In recent years, biological and molecular targeted drugs have been developed and implemented for the treatment of malignant melanoma. These drugs include vemurafenib (a selective BRAF V600 kinase inhibitor) [7], ipilimumab (an antibody preparation that targets CTLA-4 and inhibits T-cell activation) [8], and nivolumab (a mAb to PD-1) [9]. PD-1 is an inhibitory and co-stimulatory factor that is expressed on activated T cells and was first identified by Ishida et al. in 1992 as a protein that is upregulated during T cell apoptosis [10]. In this context, PD-L1 and PD-L2 are expressed in cancer cells, where they bind to PD-1 and deliver inhibitory signals to T cells. Thus, nivolumab is a novel immune checkpoint inhibitor that exerts its anti-tumor effect by inducing PD-L1/PD-L2 binding and maintaining T cell function [11]. Clinical studies have confirmed that nivolumab is effective for treating malignant melanoma [12, 13], as it provides a 1-year survival rate of 72.9%, compared with 42.1% among patients who received dacarbazine monotherapy [4]. Currently, nivolumab therapy is indicated for patients who are refractory to conventional chemotherapy, which includes dacarbazine, and is typically administered intravenously at a dose of 2 mg/kg every 3 weeks. Treatment time with nivolumab is short because it does not require premedication to prevent the occurrence of nausea, and outpatient treatment is available, as no serious cytopenia occurs after treatment.

**Fig. 3:**
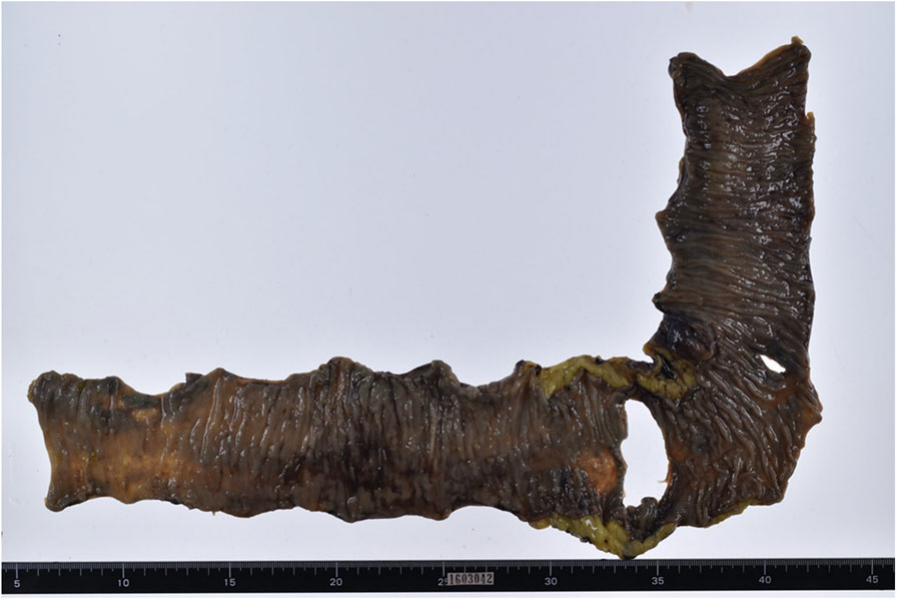
Macroscopic findings

Nivolumab has several side effects, which are often considered relatively mild (e.g., malaise, pruritus, and nausea) [4, 5, 12, 13]. However, nivolumab is also associated with several severe side effects, which include interstitial pulmonary disease, liver dysfunction, thyroid deficiency, diarrhea, colitis, and infusion reactions [4, 13]. To the best of our knowledge, this is the first English report on a case of intestinal perforation after nivolumab therapy. The pathological mechanism of immunoreaction resulting in intestinal perforation is similar to that of an inflammatory reaction [14]. In a previous report on inflammatory colitis with metastatic melanoma, colonoscopic biopsy specimens demonstrated interstitial lymphoid infiltration after administration of anti-programmed death-1 antibody [15]. In the resected specimen obtained from our patient, we observed similar inflammatory findings (Fig. 3). An immunologic reaction may have a potential to influence intestinal perforation, and in the present case, we cannot deny that an immunologic reaction influenced the development of intestinal perforation. We consider that the perforation in our case was associated with the autoimmune mechanism of nivolumab, which would indicate that this drug has unique properties that differentiate it from other anti-tumor drugs. However, the mechanism of gastrointestinal perforation due to nivolumab is not understood, and there is no past evidence regarding this. While it has been previously reported that using nivolumab could cause colitis, there is also the risk that the gastrointestinal perforation could appear during treatment; therefore, physicians should be aware of the risk of gastrointestinal perforation when using nivolumab.

Nivolumab therapy has potential efficacy for malignant melanoma refractory to existing pharmaceutical therapies, such as in the present case. Moreover, nivolumab therapy could improve overall survival and quality of life among patients with malignant melanoma, and is expected to become a central or combination therapy for patients with unresectable malignant melanoma. However, nivolumab therapy can cause serious side effects (e.g., intestinal perforation in the present case), and patients who receive nivolumab therapy should be monitored for the onset of possible side effects. Nevertheless, the accumulation of additional cases or large-scale studies are needed to validate our findings.

## Conclusions

We treated a patient with a malignant melanoma, who subsequently developed intestinal perforation due to nivolumab therapy.

## Abbreviations

CTLA-4: Cytotoxic T-lymphocyte antigen-4
PD-1: Programmed death-1
PD-L1: Programmed death-ligand 1
